# Early-Onset Colorectal Cancer: When to Include Cowden Syndrome in Your Differential Diagnosis

**DOI:** 10.14309/crj.0000000000001643

**Published:** 2025-03-12

**Authors:** Joshua Morny, Anna Zakas, Samuel Koebe, Jennifer M. Weiss

**Affiliations:** 1 Department of Medicine, University of Wisconsin School of Medicine and Public Health, Madison, WI; 2 Oncology Genetics, University of Wisconsin Carbone Cancer Center, UW Health, Madison, WI; 3 Department of Radiology, University of Wisconsin School of Medicine and Public Health, Madison, WI; 4 Division of Gastroenterology and Hepatology, University of Wisconsin School of Medicine and Public Health, Madison, WI

**Keywords:** early-onset colorectal cancer, Cowden syndrome, hereditary colon cancer syndromes

## CASE REPORT

**Figure 1.**
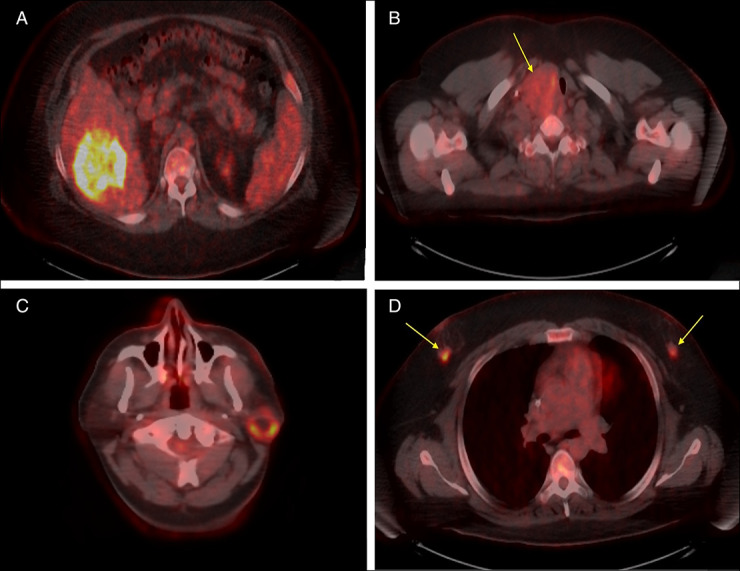
Positron emission tomography-computed tomography images of (A) solitary liver mass, (B) enlarged right thyroid gland, (C) left parotid gland, and (D) bilateral breast tissue.

**Figure 2.**
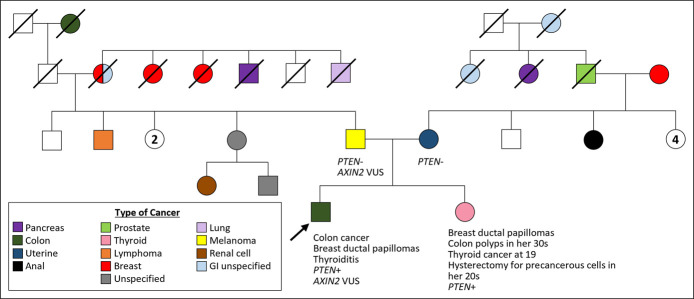
Four generation pedigree showing multiple cancers on both sides of the family, as well as germline genetic test results for immediate family members. The fact that neither parent testing positive for the *PTEN* gene mutation raises the possibility of gonadal mosaicism in either his mother or father. GI, gastrointestinal; VUS, variant of uncertain significance.

A 35-year-old man with autism and thyroiditis presented with hematochezia on anticoagulation. Endoscopy showed esophageal glycogenic acanthosis and a sigmoid mass concerning for adenocarcinoma. Staging positron emission tomography-computed tomography showed intense uptake in multiple organs, plus the sigmoid mass and bilateral adrenal myelolipomas (Figure 1). Biopsies confirmed metastatic adenocarcinoma in the liver with intact expression of the 4 DNA mismatch repair proteins, histiocytes with giant cells in the parotid, and breast intraductal papillomatosis. Four-generation pedigree was obtained (Figure 2). Multigene panel testing identified a heterozygous pathogenic mutation in the *PTEN* gene c.388C>T consistent with Cowden syndrome (CS). Unfortunately, he developed progression of metastatic disease and was placed in hospice. One in 5 individuals with early-onset colorectal cancer (EOCRC) has a hereditary cancer syndrome.^1^ Cowden syndrome is a rare cause of EOCRC. The National Comprehensive Cancer Network Clinical Practice Guidelines in Oncology recommends multigene panel testing for colorectal cancer risk-associated genes in all patients with EOCRC.^2^ CS should be considered if staging workup shows thyroid enlargement, nodular breast tissue, parotid gland involvement, and adrenal myelolipomas. The autism spectrum disorder, thyroiditis, and esophageal glycogenic acanthosis in our patient were also consistent with CS. Identification of CS has significant implications for the patient, as well as at-risk family.^3^

## DISCLOSURES

Author contributions: J. Morny: conceptualization of the case report, drafting of the manuscript, and reviewed the final version of the manuscript. S. Koebe: provided the images. A. Zakas and J. Weiss: reviewed the final version of the manuscript and provided expert input. J. Weiss accepts full responsibility for the conduct of the study and is the article guarantor.

Financial disclosure: None to report.

Previous presentation: This case report was previously presented at the American College of Gastroenterology 2023 Annual Meeting; October 20-25, 2023; Vancouver, British Columbia, Canada.

Informed consent was obtained for this case report.
